# Redirection of the Reaction Specificity of a Thermophilic Acetolactate Synthase toward Acetaldehyde Formation

**DOI:** 10.1371/journal.pone.0146146

**Published:** 2016-01-05

**Authors:** Maria Cheng, Hayato Yoshiyasu, Kenji Okano, Hisao Ohtake, Kohsuke Honda

**Affiliations:** 1 Department of Biotechnology, Graduate School of Engineering, Osaka University, 2–1 Yamadaoka, Suita, Osaka 565–0871, Japan; 2 Core Research for Evolutional Science and Technology (CREST), Japan Science and Technology Agency (JST), 7 Gobancho, Chiyoda-ku, Tokyo 102–0076, Japan; Centro de Biología Molecular Severo Ochoa (CSIC-UAM), SPAIN

## Abstract

Acetolactate synthase and pyruvate decarboxylase are thiamine pyrophosphate-dependent enzymes that convert pyruvate into acetolactate and acetaldehyde, respectively. Although the former are encoded in the genomes of many thermophiles and hyperthermophiles, the latter has been found only in mesophilic organisms. In this study, the reaction specificity of acetolactate synthase from *Thermus thermophilus* was redirected to catalyze acetaldehyde formation to develop a thermophilic pyruvate decarboxylase. Error-prone PCR and mutant library screening led to the identification of a quadruple mutant with 3.1-fold higher acetaldehyde-forming activity than the wild-type. Site-directed mutagenesis experiments revealed that the increased activity of the mutant was due to H474R amino acid substitution, which likely generated two new hydrogen bonds near the thiamine pyrophosphate-binding site. These hydrogen bonds might result in the better accessibility of H^+^ to the substrate-cofactor-enzyme intermediate and a shift in the reaction specificity of the enzyme.

## Introduction

Enzymes have been recognized as a powerful tool in chemical manufacturing processes and are replacing conventional metallo- and organocatalysts [1]. Thermostable enzymes, in particular, are attracting much attention owing to their inherent stability and compatibility with high-temperature and harsh industrial processes, and therefore bioprospecting effort has been devoted to obtain novel thermostable enzymes with desired catalytic properties [2,3]. To date, there have been two major strategies to obtain thermostable enzymes. The first is by increasing the thermostability of mesophilic enzymes by random mutation [4–8] and by rational design based on known stabilization mechanisms [9–14]. The second strategy is by mining thermophilic and hyperthermophilic microorganisms for their indigenous enzymes. Enzymes from these (hyper)thermophiles have been reported to display a higher tolerance not only to high temperatures but also to denaturants, such as detergents and organic solvents, than their mesophilic counterparts and are therefore of interest in chemical manufacturing [15]. Studies on the stabilization mechanisms of these thermophilic enzymes have made it possible to design enzymes with higher thermostability [16–18].

In this study, we propose an alternative approach to obtaining a thermostable enzyme. Pyruvate decarboxylases (EC 4.1.1.1) are the class of enzymes catalyzing the non-oxidative decarboxylation of pyruvate to acetaldehyde, which serves as a primary precursor for the production of ethanol and acetyl-CoA. Pyruvate decarboxylases and their genes are widely distributed in yeast, fungi, and higher plants but are relatively rare in prokaryotes [19]. Among the prokaryotic pyruvate decarboxylases, those from mesophilic bacteria, including *Zymomonas mobilis* [20] and *Acetobacter pasteurianus* [21], have been well characterized. However, BLAST searches of the fully sequenced genomes of (hyper)thermophiles gave no hits when the amino acid sequences of these mesophilic pyruvate decarboxylases were used as queries [19,22]. On the other hand, database searches have revealed that many thermophilic enzymes, which are annotated as acetolactate synthase (EC 2.2.1.6), share a certain level of similarity with pyruvate decarboxylases.

**Fig 1:**
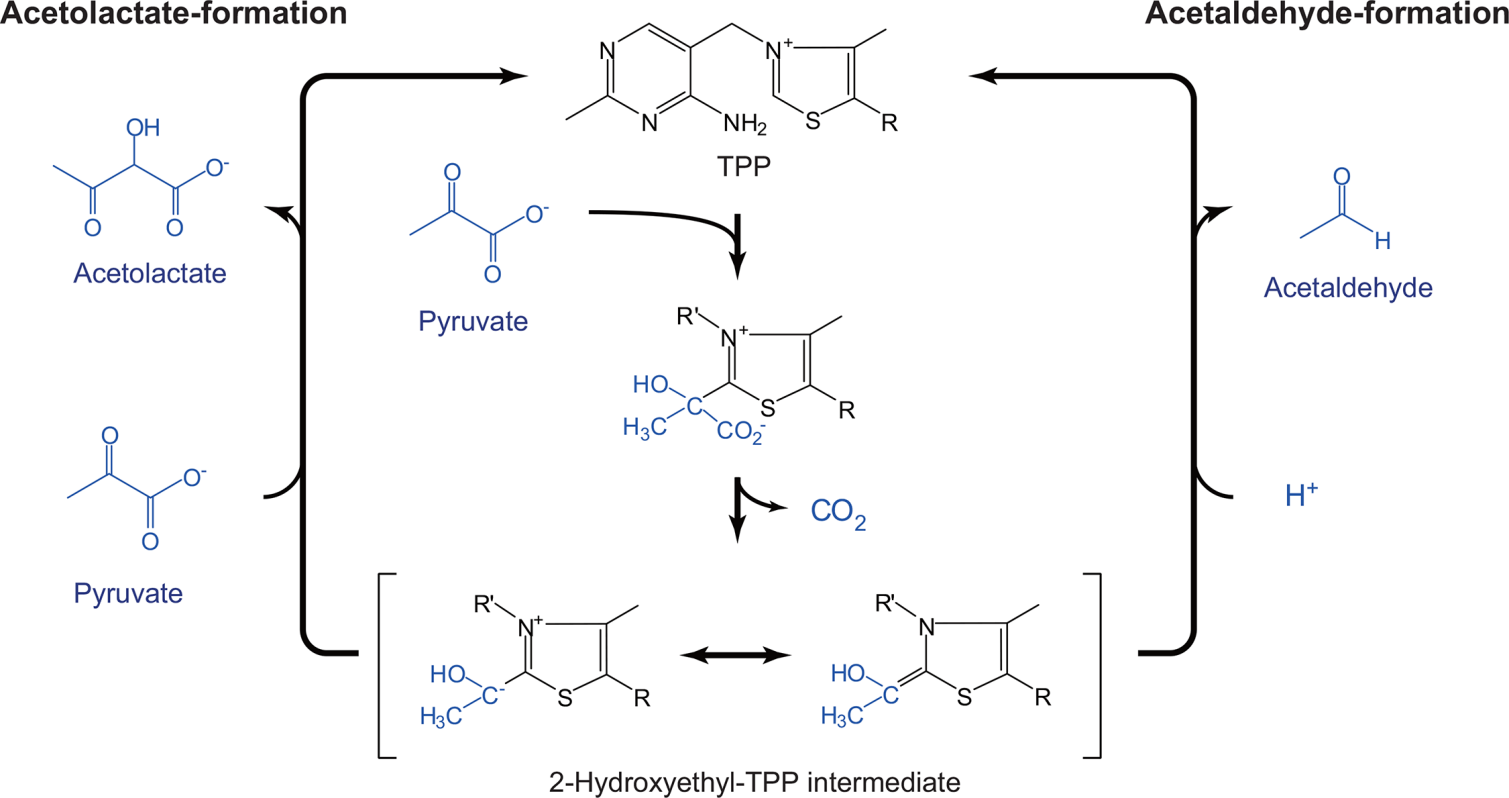
Schematic representation of the catalytic cycle of acetolactate synthase and pyruvate decarboxylase.

Acetolactate synthase and pyruvate decarboxylase are both thiamin pyrophosphate (TPP)-dependent enzymes that use pyruvate as a substrate, but they produce different products (Fig 1). Whereas pyruvate decarboxylase catalyzes the non-oxidative decarboxylation of pyruvate to acetaldehyde [23–26], acetolactate synthase, which is involved in the biosynthesis of branched amino acids (Val, Leu, Ile), catalyzes the carboligation between two pyruvate molecules to form an acetolactate molecule and carbon dioxide [23,27–29]. Despite differences in their apparent enzymatic function, the amino acid sequence comparison of several acetolactate synthases and pyruvate decarboxylases showed that they have comparable sequence similarity as well as conserved amino acids (S1 Fig). In addition, the conversion of pyruvate by acetolactate synthase and pyruvate decarboxylase proceeds via the formation of a common substrate-cofactor-enzyme complex (Fig 1) [25,26,30–32]. The carbonyl addition of pyruvate to TPP yields a predecarboxylation intermediate followed by the elimination of carbon dioxide, resulting in the formation of a central and highly reactive intermediate, 2-hydroxyethyl-TPP. In acetolactate synthase, the carboligation between 2-hydroxyethyl-TPP and the second pyruvate molecule leads to the liberation of the reaction product, acetolactate, and the catalytic cycle is completed. On the other hand, protonation of the 2-hydroxyethyl-TPP intermediate preferably occurs in pyruvate decarboxylase, yielding acetaldehyde as a reaction product.

On the basis of these structural and functional similarities, we aimed to generate a thermostable pyruvate decarboxylase through the alteration of the reaction specificity of a thermophilic acetolactate synthase by random mutagenesis.

## Materials and Methods

### Gene cloning and construction of mutant library

The expression vector for *A*. *pasteurianus* pyruvate decarboxylase (*Ap*PDC) was constructed as described previously [22]. The plasmid encoding the acetolactate synthase large subunit (*Tt*ALS; Genbank accession number, YP_144479.1) was obtained from the RIKEN *Thermus thermophilus* HB8 expression plasmid set [33] and used as a gene source. The *Tt*ALS gene was amplified by PCR using ALS-F and -R primers (Table 1), gel-purified, and digested with *Eco*RI and *Hin*dIII. The DNA fragment was introduced to the corresponding restriction site of pUC-18 (designated as pUC-*Tt*ALS) and then transformed into *Escherichia coli* JM109.

**Table 1:** Oligonucleotide primers used in this study.

Primer	Sequence (5´-3´)	Purpose
ALS-F	TCGAATTC^a^GAAGGGAGCGGAGGCACTTTTA	Vector construction
ALS-R	CCAAGCTT^b^TCACGCCCCCACCTCCTCCT	Vector construction
M13-F	TGTAAAACGACGGCCAGT	Error-prone PCR
M13-R	CAGGAAACAGCTATGAC	Error-prone PCR
Y35N-F	GCCCTCA^c^ACGACAGCCCCATCCGCCAC	Site-directed mutagenesis
Y35N-R	GCTGTCGTT^c^GAGGGCGTCGTAGGTGGG	Site-directed mutagenesis
K139R-F	GGTGGTGAG^c^GGAGGCCTTCCACATCGC	Site-directed mutagenesis
K139R-R	GCCTCCC^c^TCACCACCCGGGGGATCTCG	Site-directed mutagenesis
V172A-F	TTGACGC^c^GAAGCTGGACCTCCCCGGGT	Site-directed mutagenesis
V172A-R	CCAGCTTCG^c^CGTCAAAGCTCCCCGTGA	Site-directed mutagenesis
H474R-F	TCTTCCG^c^CGCCAAGCGCTACAGCGAGG	Site-directed mutagenesis
H474R-R	GCTTGGCGC^c^GGAAGAGGTCCTGCCACT	Site-directed mutagenesis

^a^ *Eco*RI restriction sight is underlined.

^b^ *Hin*dIII restriction sight is underlined.

^c^ Substituted nucleotides are underlined.

Random mutagenesis was introduced to the *Tt*ALS gene by error-prone PCR. The PCR mixture contained GoTaq Green Master Mix (Promega KK, Tokyo, Japan), 0.2 mM M13-F and M13-R primers (Table 1), 0.1 mM MnCl_2_, and approximately 10 ng of pUC-*Tt*ALS in a total volume of 12.5 μl. PCR was carried out with 95°C preheating for 5 min, 30 cycles of 95°C for 30 s, 55°C for 1 min, 72°C for 1 min 40 s, followed by an elongation step at 72°C for 7 min. This operation resulted in the 1–4 mutation points in the 1,689-bp long *Tt*ALS gene. PCR products were purified, restricted, ligated to pUC-18, and transformed into *E*. *coli* JM109, as described above. Blue/white selection on an LB agar supplemented with 100 μg ml^-1^ ampicillin, 40 μg ml^-1^ 5-bromo-4-chloro-3 -indolyl-β-D-galactoside (X-gal, Wako Pure Chemical, Osaka, Japan), and 0.1 mM isopropyl β-D-1-thiogalactopyranoside (IPTG, Wako Pure Chemical) was performed to select positive transformants. White colonies were picked and stored as a mutant library.

### Site-directed mutagenesis

PrimeStar mutagenesis kit (Takara Bio, Ohtsu, Japan) was used to create single-amino-acid substituted mutants (Y35N, K139R, V172A, and H474R). PCR was performed in accordance with the manufacturer’s instructions using pUC-*Tt*ALS as a template DNA and the primers listed in Table 1. The DNA sequence was confirmed with the 3130 Genetic Analyzer (Applied Biosystems, Foster City, CA).

### Mutant library screening

A colorimetric screening system was developed to evaluate the acetaldehyde-forming activity of mutant enzymes. The reaction was coupled with NAD^+^-dependent acetaldehyde dehydrogenase from *T*. *thermophilus* HB8 (*Tt*ALDH; Genbank accession number, YP_145486.1) [22]. The *Tt*ALDH gene was obtained from the RIKEN plasmid set and the enzyme was prepared in *E*. *coli* Rosetta 2 (DE3) as described elsewhere [22].

Clones in the mutant *Tt*ALS library were aerobically cultivated in a 96-deep-well plate at 30°C for 15 hours. Each well contained 500 μl of LB medium supplemented with 100 μg ml^-1^ ampicillin and 0.1 mM IPTG. Cells were pelleted by centrifugation at 1,500 × *g* and 4°C, for 10 min. The average weight of the cell pellet in each well was calculated from the increase in the total weight of the multiwell plate, and the cells were resuspended in 50 mM potassium phosphate buffer (pH 7.0) containing 10 mM MgCl_2_⋅6H_2_O at an average cell concentration of 50 mg wet cells ml^-1^. An aliquot (100 μl) of the cell suspension was transferred to a 200 μl PCR tube and heated at 70°C for 30 min with T3000 thermocycler (Biometra, Göttingen, Germany). The cell suspension was then mixed with a 100 μl reaction mixture consisting of 5 mM sodium pyruvate, 12 μM 1-methoxy-5-methylphenazinium methylsulfate (1-methoxy PMS, Dojindo, Kumamoto, Japan), 0.3 mM 3-[4,5-dimethylthiazol-2-yl]-2,5-diphenyltetrazolium bromide (MTT, Nacalai Tesque, Kyoto, Japan), 2 mM NAD^+^ (Oriental Yeast, Tokyo, Japan), 0.2 mM thiamine pyrophosphate (TPP; Wako Pure Chemical), and 100 mg wet cells ml^-1^ of the heat-treated (70°C for 30 min) cell suspension of *E*. *coli* with *Tt*ALDH [22]. The mixture was incubated at 60°C for 30 min and the formation of MTT formazan was visually monitored.

### Enzyme preparation

*E*. *coli* cells having the wild-type and mutant *Tt*ALS were aerobically cultivated at 37°C in a 500 ml Erlenmeyer flask containing LB medium supplemented with 100 μg ml^-1^ ampicillin. Gene expression was induced by adding 0.1 mM IPTG in the late-log phase. The cells were harvested by centrifugation and resuspended in 20 mM Tris-HCl (pH 8.0) at a cell concentration of 200 mg wet cells ml^-1^. The cells were disrupted by ultrasonication and centrifuged to remove the cell debris. The soluble fraction was collected as a crude lysate and the total protein concentration was measured with Bio-Rad protein assay kit II (Bio-Rad, Hercules, CA). The crude lysate was then heated at 70°C for 30 min and centrifuged to remove denatured proteins. The resulting supernatant was used as a heat-treated crude extract. The heat-treated extract of *E*. *coli* with *Ap*PDC was prepared in the same manner except that the heat treatment was done at 50°C for 30 min owing to the relatively low thermal stability of the enzyme.

### Enzyme assays

The acetaldehyde-forming activity of the enzyme was determined by coupling with *Tt*ALDH. The reaction mixture was composed of 50 mM potassium phosphate buffer (pH7.0), 10 mM sodium pyruvate, 240 μM 1-methoxy PMS, 6 mM 2-(4-iodophenyl)-3-(4-nitrophenyl)-5-(2,4-disulfophenyl)-2*H*-tetrazolium (WST-1; Dojindo), 0.67 mM NAD^+^, 0.1 mM TPP, 10 mM β-mercaptoethanol, 10 mM MgCl_2_, and the heat-treated extract of *E*. *coli* with *Tt*ALDH. After preincubation at 60°C for 2 min, reaction was initiated by adding an appropriate amount of *Tt*ALS or its mutants. The reduction of WST-1 to soluble formazan was monitored at 438 nm with UV-2600 spectrophotometer (Shimadzu, Kyoto, Japan). Enzyme activity was calculated using the molar extinction coefficient of the soluble formazan (37×10^3^ cm^-1^ at 438 nm). One unit of enzyme activity was defined as the amount of enzyme required to form 1 μmol formazan per minute.

Acetolactate-forming activity was assessed by mixing the enzyme solution with 50 mM potassium phosphate buffer (pH7.0), 20 mM sodium pyruvate, 0.1 mM TPP, and 10 mM MgCl_2_. After incubation at 60°C for 30 minutes, 250 μl of the sample was taken, mixed with 50 μl of 50% H_2_SO_4_, and incubated at 37°C for 30 min to promote the decarboxylation of acetolactate to acetoin. Subsequently, 500 μl each of 0.5% creatin, 5% α-naphtol, and 2.5 M NaOH were added to the mixture and incubated at 37°C for 30 min. Acetoin concentration was determined by measuring the absorbance at 540 nm. The concentration was calculated from a standard curve obtained using serially diluted authentic acetoin (Tokyo Chemical Industry, Tokyo, Japan). One unit of enzyme activity was defined as the amount of enzyme required to form 1 μmol acetoin per minute.

### Multiple alignment and structural modeling

Multiple alignment of amino acid sequences was performed with the ClustalW2 online tool [34]. The addition of a secondary structure element and the rendering of similarities from aligned sequences were conducted with ESPript (http://espript.ibcp.fr) online tool [35]. Model structures of *Tt*ALS and its mutants were generated by SWISS-MODEL homology modeling [36]. The enzyme structure was visualized with the PyMOL Molecular Graphics System (Version 1.3, Schrödinger, LLC). The structures of the proteins were compared by the secondary structure matching (SSM) method using the Coot program [37].

## Results

### Selection and characterization of model enzymes

The protein BLAST search using *T*. *thermophilus* HB8 acetolactate synthase (*Tt*ALS) large subunit as query resulted in 90–100% sequence identity with other known thermophilic acetolactate synthases, suggesting high sequence similarities among these enzymes. Since the genomic sequence and gene-expression plasmid set of *T*. *thermophilus* HB8 are available [33], enzymes from this organism are more feasible compared to those from other thermophilic sources. In addition, when compared to amino acid sequence of several pyruvate decarboxylases, *Tt*ALS also showed relatively high similarities (S1 Fig). Considering these facts, we chose *Tt*ALS as a model thermophilic acetolactate synthase.

The reaction specificity of *Tt*ALS was assessed and compared with that of a bacterial pyruvate decarboxylase from *A*. *pasteurianus* (*Ap*PDC) (Table 2). *Ap*PDC exhibited not only physiological activity (acetaldehyde formation) but also a significant acetolactate-forming activity. In contrast, the reaction catalyzed by *Tt*ALS was more specific to acetolactate formation.

**Table 2:** Specific enzyme activities.

Enzyme	Specific activity (x 10^−3^ U mg^-1^ total protein)^a^^,^^b^		Ratio^c^
	Acetolactate formation	Acetaldehyde formation	
*Ap*PDC ^d^	10.8 ± 0.47	8.12 ± 1.8	0.76
*Tt*ALS_WT ^e^	12.6 ± 0.63	1.45 ± 0.19	0.12
*Tt*ALS_quadruple ^e^	7.96 ± 1.5	4.51 ± 0.42	0.57
*Tt*ALS_Y35N ^e^	10.6 ± 0.46	2.00 ± 0.28	0.19
*Tt*ALS_K139R ^e^	12.2 ± 3.0	2.23 ± 0.46	0.18
*Tt*ALS_V172A ^e^	10.2 ± 2.6	1.27 ± 0.18	0.12
*Tt*ALS_H747R ^e^	8.13 ± 2.0	5.52 ± 0.87	0.68

^a^ Specific enzyme activity was measured using the heat-treated extracts of *E*. *coli* with indicated enzymes and normalized by the protein concentration of the corresponding non-heated crude lysate. The enzyme activities were calculated by subtracting those detected in control experiments, which were conducted using the heat-treated extracts of *E*. *coli* without the expression vector.

^b^ The enzyme assays were performed at least in triplicate; mean±standard deviations are shown.

^c^ Specific acetaldehyde-forming activities were divided by the acetolactate-forming ones.

^d^ Heat-treated extract was prepared by incubating the crude lysate of the recombinant *E*. *coli* at 50°C for 30 min.

^e^ Heat-treated extracts were prepared by incubating the crude lysate of the recombinant *E*. *coli* at 70°C for 30 min.

### Mutant library screening and characterization of the positive mutant

**Fig 2:**
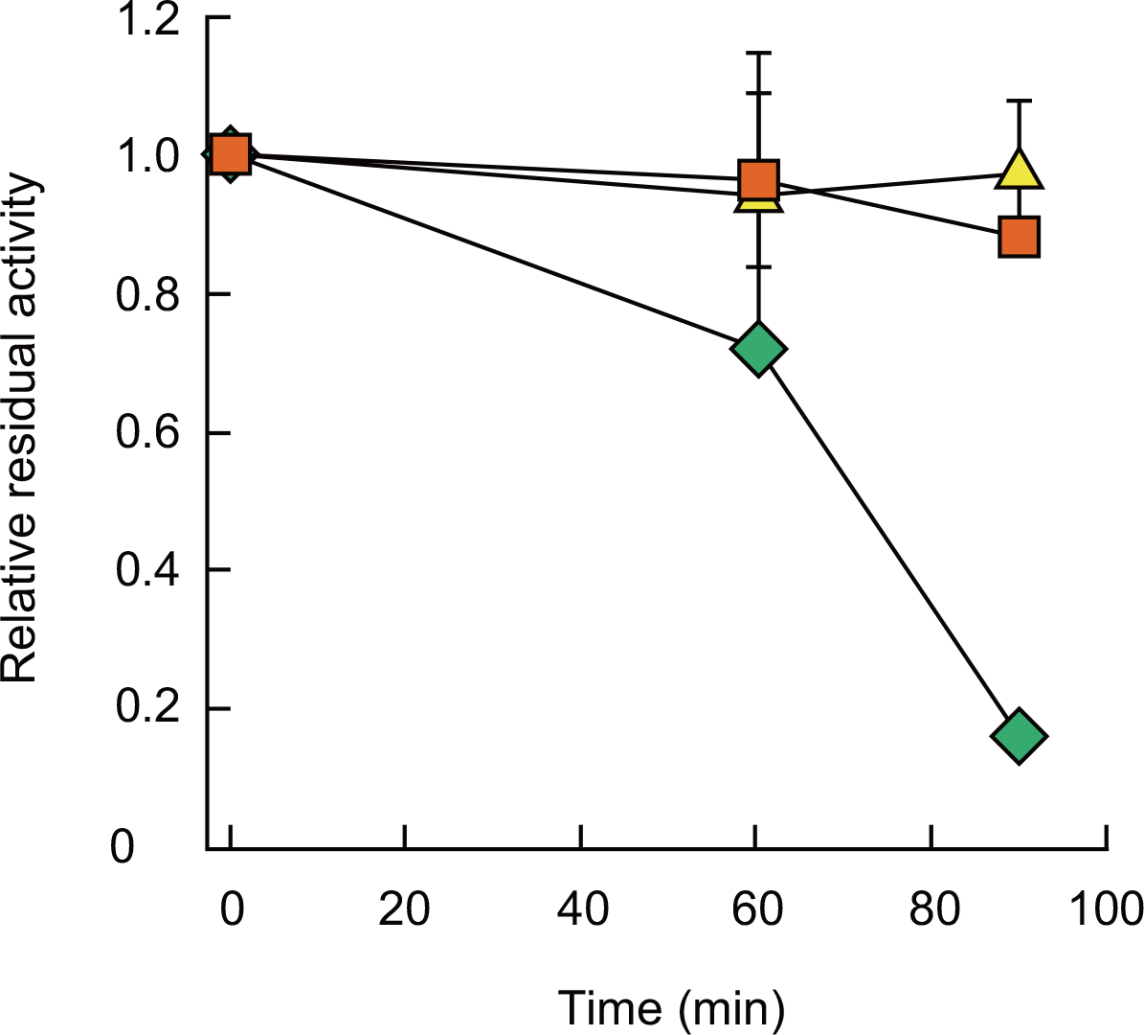
Thermal stability of *Tt*ALS_WT (red squares), *Tt*ALS_quadruple (yellow triangles), and *Ap*PDC (green diamonds). Enzyme activity was determined by measuring the acetolactate-formation activity of each enzyme after incubation at 60°C for the indicated time period. The assays were performed at least in triplicate; mean±standard deviations (error bars) are depicted.

A colorimetric, high-throughput assay system was developed to screen the *Tt*ALS-mutant library for improved acetaldehyde-forming activity. In this system, enzymatically generated acetaldehyde was further converted by another enzyme, the aldehyde dehydrogenase from *Thermus thermophilus* HB8 (*Tt*ALDH) [22], with a concomitant reduction of NAD^+^ to NADH. NADH was then used as an electron donor for the reduction of MTT to a water-insoluble formazan, which is a blue-colored compound, in the presence of an electron mediator, 1-methoxy PMS. About 5,000 transformants were screened using this system, and 12 mutants were selected for their relatively high signal intensities. The specific acetolactate-forming activities of these mutants were then spectrophotometrically assessed under standard assay conditions; and consequently, one mutant was confirmed to exhibit a 3.1-fold higher activity than that of wild-type *Tt*ALS (*Tt*ALS_WT) (Table 2). On the other hand, the acetolactate-forming activity of the mutant decreased to 63% of that of *Tt*ALS_WT. The ratio of the specific acetaldehyde-forming activity to the acetolactate-forming activity of the resulting mutant was 4.9-fold higher than that of *Tt*ALS_WT. Sequencing analysis revealed that there are four amino acid substitutions (Y35N, K139R, V172A and H474R) in the mutant; thus, it was designated as *Tt*ALS_quadruple. As expected, *Tt*ALS_quadruple exhibited a similar thermal stability to *Tt*ALS (Fig 2). Although *Ap*PDC lost more than 80% of its initial activity after the incubation at 60°C for 90 min, the wild-type and the quadruple mutant of *Tt*ALS remained almost intact upon the same treatment.

### Site-directed mutagenesis

Single-point mutants with either one of the four amino-acid substitutions found in *Tt*ALS_quadruple were constructed by site-directed mutagenesis to examine their contributions to the alteration of the reaction specificity of the enzyme. Among the resulting mutants, the H474R mutant exhibited a higher acetolactate-forming activity than the quadruple mutant (Table 2). In contrast, the reaction-specificity profiles of the other single-point mutants were not significantly different from that of *Tt*ALS_WT, demonstrating that the improved acetaldehyde-forming activity of *Tt*ALS_quadruple was largely due to the H474R mutation. The ratio of the specific acetaldehyde-forming to acetolactate-forming activity of the H474R mutant reached 0.68, approaching that of *Ap*PDC (0.76).

### Structural modeling analysis

**Fig 3:**
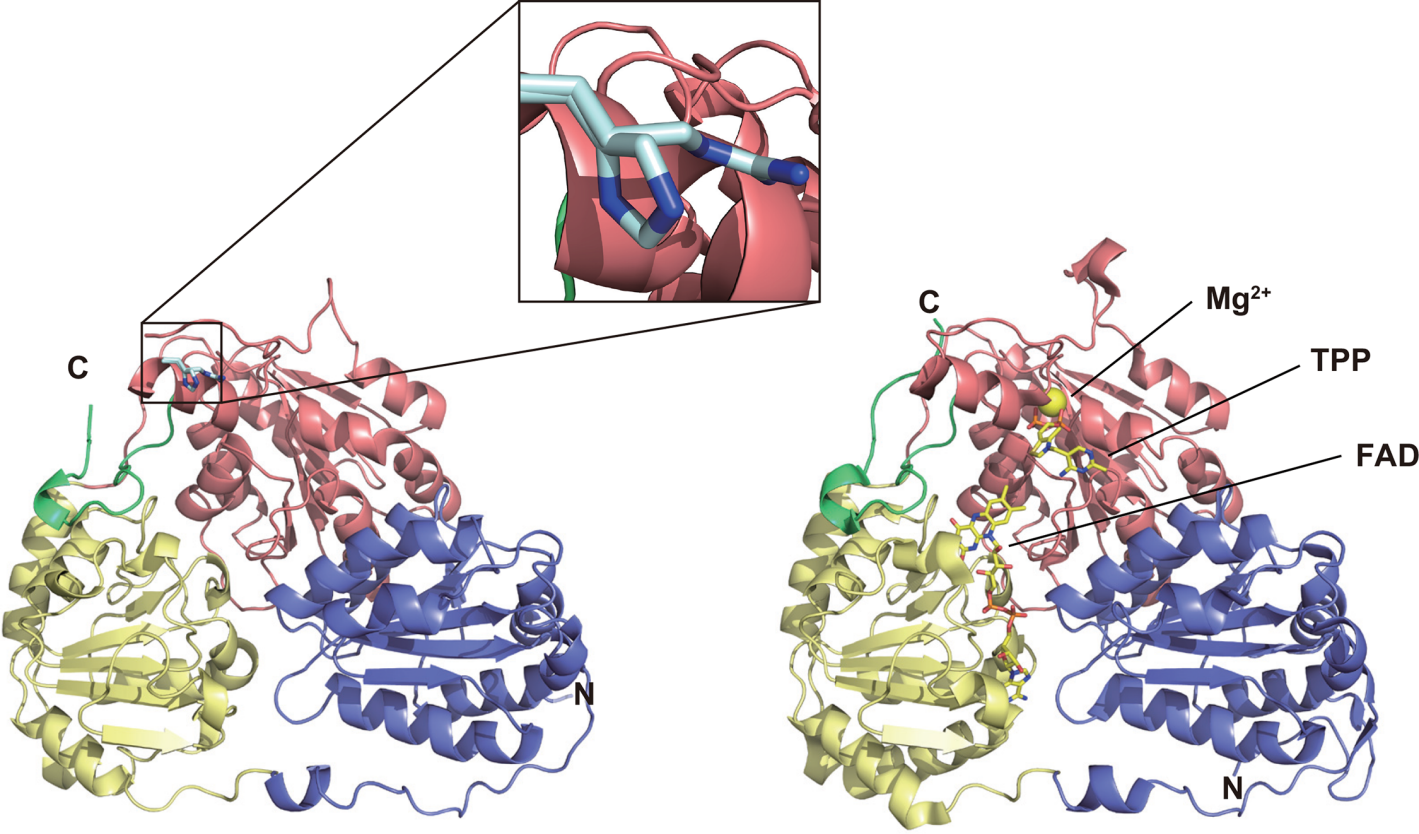
Comparison of the model structure of *Tt*ALS (left panel) and the crystal structure of *At*AHAS (right panel). N- and C-terminal of the proteins are shown by the letters N and C, respectively. The α-, β-, and γ-domains, and the C-terminal tail are shown in light blue, light yellow, pink, and light green, respectively. In the *At*AHAS structure, Mg^2+^ is shown as a yellow sphere while the backbones of other cofactors are shown in yellow with oxygen and nitrogen atoms colored red and blue, respectively. The detailed location of the H474R mutation in *Tt*ALS is shown in the inset with the backbone of the mutated residue shown in gray.

Model structures of the wild-type and the H474R mutant *Tt*ALS were built on the basis of protein homology using the SWISS MODEL program (Fig 3). The crystal structure of the acetohydroxy-acid synthase from *Arabidopsis thaliana* (*At*AHAS; PDB ID, 1Z8N), was chosen by the program as the best template among the available protein structures. The model structure of *Tt*ALS implied that both the wild-type and the H474R mutant function as a homotetramer, similarly to other structurally characterized acetolactate synthases [29,32,38]. As expected, the model revealed structural similarity between *Tt*ALS and *Ap*PDC (RMSD 2.08 Å, S2 Fig), supporting the validity of our concept of engineering *Tt*ALS to develop a thermostable pyruvate decarboxylase.

**Fig 4:**
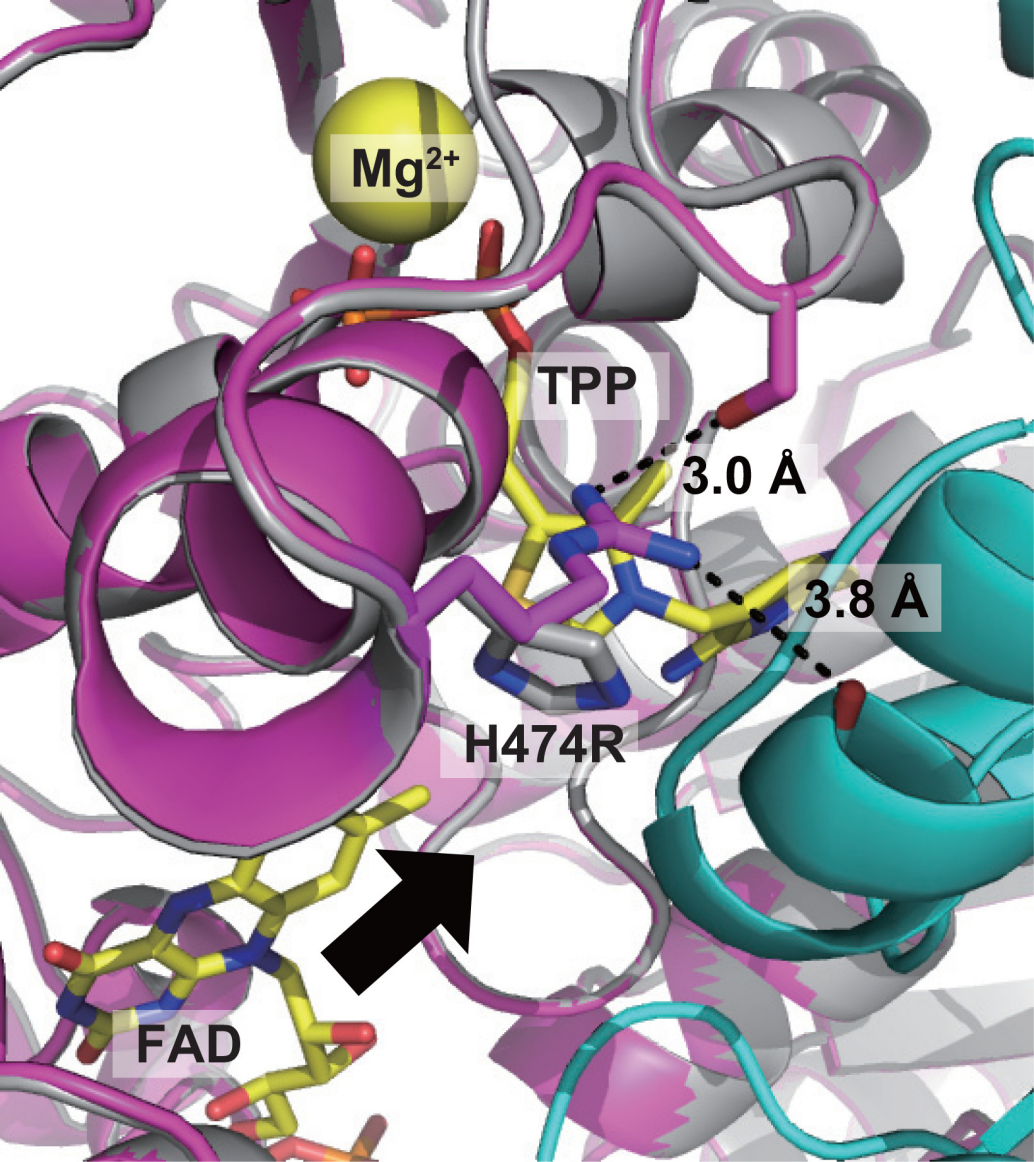
TPP binding site of the model structure of *Tt*ALS. *Tt*ALS_WT, *Tt*ALS_H474R, and the neighboring monomer are shown in gray, purple, and cyan, respectively. TPP and FAD are shown in yellow, whereas Mg^2+^ is represented as a yellow sphere. Oxygen atoms are shown in red whereas nitrogen atoms are in blue. New hydrogen bonds are formed between the side chain of H474R with the side chain of S479 and the main chain of M28, which belongs to the neighboring monomer (shown by dotted lines). The cavity through which substrates seem to access the catalytic site is indicated by a black arrow.

The model structure also showed that the H474R mutation is located at the γ-domain (Figs 3 and 4), *i*.*e*., the TPP-binding domain, of *Tt*ALS. The helix containing H474R is located on the protein surface in the interfacial area between two monomers of *Tt*ALS and forms an active site with the neighboring monomer (Fig 4). The *Tt*ALS_H474R model showed that two newly generated hydrogen bonds, which are also located on the protein surface, are present in the mutant protein. This might result in the increased hydrophilicity in the local area where TPP is bound and also make it easier for H^+^ to attack the substrate-TPP-enzyme complex intermediate in the enzymatic reaction, thus allowing the mutant to function as a pyruvate-decarboxylase-like protein.

Recently, Meyer *et al*. [31] reported the structural analysis on the transitional states of the 2-hydroxyethyl-TPP intermediate (carbanion-enamine intermediate) in TPP-dependent enzymes, leading us to another possible mechanism for the altered reaction specificity of the H474R mutant. The positive charge provided by Arg side chain might contribute to the stabilization of the negatively charged carbanion intermediate and result in the redirection of the reaction specificity of the enzyme. Previous works with *Zymomonas mobilis* pyruvate decarboxylase also demonstrated the importance of the amino acid residues in the active site of the enzyme for proper substrate binding [39,40]. Considering the position of H474R in the enzyme, it is likely that the positively charged group from Arg formed an ion pair with the carbanion intermediate and facilitated the proton addition.

## Discussion

TPP is an important cofactor involved in various types of enzyme reactions, including the decarboxylation, dehydrogenation, and carboligation of keto acids and their derivatives [23,26]. Some of them have recently attracted biotechnological interest as a catalytic module to construct an engineered metabolic pathway. Atsumi *et al*. demonstrated the non-fermentative production of isobutanol by an engineered *E*. *coli*, in which the heterologously expressed acetolactate synthase of *Bacillus subtilis* served as a key enzyme [41]. Opgenorth *et al*. reported the *in vitro* reconstitution of thermophilic pyruvate dehydrogenase complexes with different nicotinamide-cofactor specificities [42]. The reconstituted enzyme complexes were used to construct a molecular purge valve system for maintaining the intrapathway NADP^+^/NADPH balance in *in vitro* artificial pathways for polyhydroxybutyryate and isoprene production. Among TPP-dependent enzymes, pyruvate decarboxylases play a key role in fermentative production of ethanol and related compounds. *In vitro* synthesis of ethanol from glucose has been demonstrated by applying a pyruvate decarboxylase at 50°C [43]. Similarly, we constructed cofactor-balanced, oxygen-insensitive artificial pathway for the *in vitro* conversion of glucose to 1-butanol [22] and *N*-acetylglutamate [44] by employing *Ap*PDC at 50°C. Thermostable pyruvate decarboxylases would be a promising enzyme module to establish more feasible *in vitro* bioconversion systems as well as to develop an engineered thermophile for consolidated bioprocessing at high temperatures [43,45,46].

However, the number of reports on thermophilic TPP-dependent enzymes is still limited. In particular, to the best of our knowledge, there have been no reports on (hyper)thermophile-derived pyruvate decarboxylase [19,22]. Although several pyruvate ferredoxin oxidoreductases from hyperthermophilic archaea have been reported to catalyze the analogous reaction, *i*.*e*., the non-oxidative decarboxylation of pyruvate to acetaldehyde, in the absence of CoA [47,48], they are inherently oxygen-sensitive enzymes, hampering their use in biotechnological applications. This limited availability of thermophilic pyruvate-decarboxylating enzymes motivated us to develop a thermostable pyruvate decarboxylase by redirecting the reaction specificity of a functionally and structurally related thermophilic enzyme, acetolactate synthase. Through the random-mutant-library screening followed by site-directed mutagenesis experiments, we confirmed that the single-point mutant with an amino acid substitution of H747R exhibits markedly improved pyruvate-decarboxylase-like activity. However, it should be noted that the H747R mutant still exhibits a significant acetolactate-forming activity, which may hamper the selective, high-yield production of a target compound when the enzyme is applied to a biocatalytic chemical manufacturing process. Further work focusing on decreasing the acetolactate activity would be indispensable for the application of this mutant. In contrast to our study, Sergienko and Jordan demonstrated that the reaction specificity of a yeast pyruvate decarboxylase could be shifted toward the carboligating direction by the mutations at Asp28 and Glu477 residues in the catalytic center of the enzyme [49]. Similarly, the Glu473Gln mutant of *Zymomonas mobilis* pyruvate decarboxylase was shown to catalyze an enantio-selective carboligation between pyruvate and an aromatic aldehyde and was applied to the asymmetric production of (*R*)-phenylacetyl carbinol [30]. In fact, the sequence alignment revealed that these amino acid residues are well conserved among pyruvate decarboxylases but not among acetolactate synthases (S1 Fig), suggesting that alteration of the corresponding residues of *Tt*ALS (*i*.*e*., Gly37 and Val487) may lead to the development of a mutant enzyme with further improved specificity to pyruvate decarboxylation.

## Supporting Information

S1 Fig Multiple sequence alignment of acetolactate synthases and pyruvate decarboxylases. Amino acid sequences of *Arabidopsis thaliana* acetohydroxyacid synthase (AtAHAS; PDB ID, 1Z8N) and *Thermus thermophilus* HB8 acetolactate synthase (TtALS) are aligned with those of acetolactate synthases from *Klebsiella pneumonia* (KpALS; PDB ID, 1OZG) and *Bacillus subtilis* (BsALS; PDB ID, 4RJJ), and pyruvate decarboxylases from *Zymomonas mobilis* (ZmPDC; PDB ID, 2WVA), *Acetobacter pasteurianus* (ApPDC; PDB ID, 2VBI), and *Saccharomyces cerevisiae* (ScPDC; PDB ID, 1PVD). Symbols above the alignment represent the structure of AtAHAS. α-Helices and 310-helices (η) are indicated by curved lines. Black arrows and TT letters represent β-strands and β-turns, respectively. Black dots above the AtAHAS sequence mark the sequence every 10 residues. Similar residues in the alignment are shown in blue boxes, while those printed in white on red are conserved residues. The position of H474 in the TtALS sequence is indicated by a red arrow. The positions of D28 and E477 in ScPDC, whose substitutions caused the alteration of the reaction specificity of the enzyme [49], are indicated by green asterisks.
(PDF)

S2 Fig Superimposition of *Tt*ALS model structure with the crystal structure of *Ap*PDC (PDB ID 2VBI). The *Tt*ALS model structure is colored purple whereas *Ap*PDC is shown in green.
(PDF)
