# Supplementary figures and images for: Redirection of the Reaction Specificity of a Thermophilic Acetolactate Synthase toward Acetaldehyde Formation
Source: PLoS One. 2016 Jan 5;11(1):e0146146. doi: 10.1371/journal.pone.0146146 (PMC4701669; doi:10.1371/journal.pone.0146146)

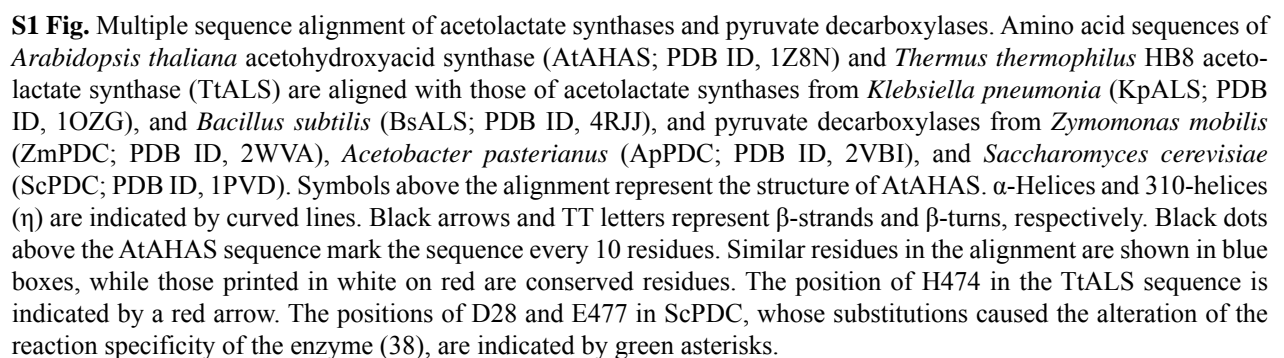

Supplement: S1 Fig — Amino acid sequences of Arabidopsis thaliana acetohydroxyacid synthase (AtAHAS; PDB ID, 1Z8N) and Thermus thermophilus HB8 acetolactate synthase (TtALS) are aligned with those of acetolactate synthases from Klebsiella pneumonia (KpALS; PDB ID, 1OZG) and Bacillus subtilis (BsALS; PDB ID, 4RJJ), and pyruvate decarboxylases from Zymomonas mobilis (ZmPDC; PDB ID, 2WVA), Acetobacter pasteurianus (ApPDC; PDB ID, 2VBI), and Saccharomyces cerevisiae (ScPDC; PDB ID, 1PVD). Symbols above the alignment represent the structure of AtAHAS. α-Helices and 310-helices (η) are indicated by curved lines. Black arrows and TT letters represent β-strands and β-turns, respectively. Black dots above the AtAHAS sequence mark the sequence every 10 residues. Similar residues in the alignment are shown in blue boxes, while those printed in white on red are conserved residues. The position of H474 in the TtALS sequence is indicated by a red arrow. The positions of D28 and E477 in ScPDC, whose substitutions caused the alteration of the reaction specificity of the enzyme [49], are indicated by green asterisks. (PDF) [file pone.0146146.s001.pdf]
