# Supplementary material for: Redirection of the Reaction Specificity of a Thermophilic Acetolactate Synthase toward Acetaldehyde Formation
Source: PLoS One. 2016 Jan 5;11(1):e0146146. doi: 10.1371/journal.pone.0146146 (PMC4701669; doi:10.1371/journal.pone.0146146)
Supplement: S2 Fig — The TtALS model structure is colored purple whereas ApPDC is shown in green. (PDF) [file pone.0146146.s002.pdf]

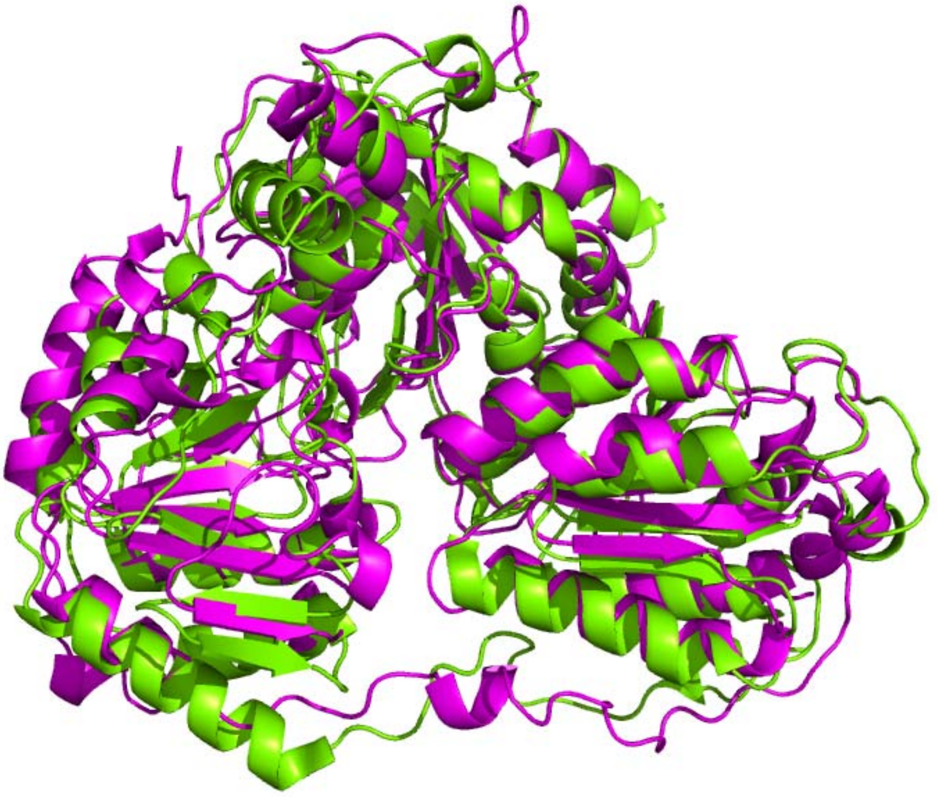

S2 Fig. Superimposition of *Tt*ALS model structure with the crystal structure of *Ap*PDC (PDB ID 2VBI). The *Tt*ALS model structure is colored purple whereas *Ap*PDC is shown in green.
